# Increased B cell activation is present in *JAK2*V617F-mutated, *CALR*-mutated and triple-negative essential thrombocythemia

**DOI:** 10.18632/oncotarget.16381

**Published:** 2017-03-18

**Authors:** Ken-Hong Lim, Caleb Gon-Shen Chen, Yu-Cheng Chang, Yi-Hao Chiang, Chen-Wei Kao, Wei-Ting Wang, Chiao-Yi Chang, Ling Huang, Ching-Sung Lin, Chun-Chia Cheng, Hung-I Cheng, Nai-Wen Su, Johnson Lin, Yi-Fang Chang, Ming-Chih Chang, Ruey-Kuen Hsieh, Huan-Chau Lin, Yuan-Yeh Kuo

**Affiliations:** ^1^ Graduate Institute of Oncology, National Taiwan University College of Medicine, Taipei, Taiwan; ^2^ Department of Internal Medicine, Division of Hematology and Oncology, MacKay Memorial Hospital, Taipei, Taiwan; ^3^ Department of Medical Research, Laboratory of Good Clinical Research Center, MacKay Memorial Hospital, Tamsui District, New Taipei City, Taiwan; ^4^ Department of Medicine, MacKay Medical College, New Taipei City, Taiwan; ^5^ Institute of Molecular and Cellular Biology, National Tsing-Hua University, Hsinchu, Taiwan; ^6^ Department of Internal Medicine, Division of Hematology and Oncology, MacKay Memorial Hospital, Hsinchu, Taiwan

**Keywords:** B cell, CALR, essential thrombocythemia, immune

## Abstract

Essential thrombocythemia (ET) is a *BCL-ABL1*-negative myeloproliferative neoplasm. We have reported that increased activated B cells can facilitate platelet production mediated by cytokines regardless *JAK2* mutational status in ET. Recently, *calreticulin* (*CALR*) mutations were discovered in ~30% *JAK2*/*MPL-*unmutated ET and primary myelofibrosis. Here we sought to screen for *CALR* mutations and to evaluate B cell immune profiles in a cohort of adult Taiwanese ET patients. B cell populations, granulocytes/monocytes membrane-bound B cell-activating factor (mBAFF) levels, B cells toll-like receptor 4 (TLR4) expression and intracellular levels of interleukin (IL)-1β/IL-6 and the expression of CD69, CD80, and CD86 were quantified by flow cytometry. Serum BAFF concentration was measured by ELISA. 48 healthy adults were used for comparison. 19 (35.2%) of 54 ET patients harbored 8 types of *CALR* exon 9 mutations including 4 (7.4%) patients with concomitant *JAK2*V617F mutations. Compared to *JAK2*V617F mutation, *CALR* mutations correlated with younger age at diagnosis (*p*=0.04), higher platelet count (*p*=0.004), lower hemoglobin level (*p*=0.013) and lower leukocyte count (*p*=0.013). Multivariate analysis adjusted for age, sex, follow-up period and hematological parameters confirmed that increased activated B cells were universally present in *JAK2*-mutated, *CALR*-mutated and triple-negative ET patients when compared to healthy adults. *JAK2*- and *CALR*-mutated ET have significantly higher fraction of B cells with TLR4 expression when compared to triple-negative ET (*p*=0.019 and 0.02, respectively). *CALR*-mutated ET had significantly higher number of CD69-positive activated B cells when compared to triple-negative ET (*p*=0.035). In conclusion, increased B cell activation is present in ET patients across different mutational subgroups.

## INTRODUCTION

Essential thrombocythemia (ET) is a *BCL-ABL1*-negative myeloproliferative neoplasm (MPN), and is characterized by increased number of mature megakaryocytes (MKs) in the bone marrow and sustained thrombocytosis in the peripheral blood [1]. ET is associated with an increased risk of hemorrhagic and thrombotic complications and leukemic transformation [1]. Most ET patients can have a normal life expectancy but some may encounter serious events during their disease course. In 2005, the *JAK2*V617F mutation was discovered in MPNs including 50-60% patients with ET and primary myelofibrosis (PMF) [2–5]. *JAK2*V617F mutation plays an important role in cytokine-independent hematopoietic stem cells (HSCs) proliferation in MPNs. Also, hypersensitivity of hematopoietic cells to cytokines stimulation is noted in MPNs through the interaction between *JAK2*V617F mutation and various cytokine receptors [6]. Recently, a high frequency of *calreticulin* (*CALR*) mutations was discovered in *JAK2*/*MPL*-unmutated ET and PMF [7–9]. We and others have reported that *CALR* mutations are associated with distinct clinical characteristics including higher platelet counts, lower leukocyte counts and hemoglobin levels, and a lower thrombosis risk when compared to *JAK2*-mutated ET patients [7–11]. Using *in vitro* and/or *in vivo* models, we and others have recently reported that mutant *CALR* can activate JAK-STAT signaling pathway through an MPL-dependent mechanism to mediate pathogenic thrombopoiesis [12–18].

CALR is a 46-kDa Ca2+ binding chaperone protein located in the endoplasmic reticulum, but it can also localize to cell surface and accumulate in extracellular compartments [19]. In addition to ensuring proper protein and glycoprotein folding within the lumen of endoplasmic reticulum, CALR was also found to involve the immune response to pre-apoptotic cancer cells, and early cell surface exposure of CALR was followed by expression and release of heat-shock proteins (e.g. HSP70), and high-mobility group I (HMGB1) protein [20]. Recombinant *CALR* fragment was shown to exhibit potent stimulatory activities against B cells [21, 22]. Recently, we reported that activated B cells are increased in ET patients, and can facilitate platelet production mediated by cytokines, such as interleukin (IL)-1β and IL-6 regardless *JAK2*V617F mutational status [23]. We found that increased production of B cell-activating factor (BAFF) by granulocytes and monocytes up-regulates toll-like receptor 4 (TLR4) expression on B cells and promotes B cell activation in ET patients. Consequently, these activated B cells play a pathogenic role in augmenting thrombocytosis by producing IL-1β and IL-6 in ET patients through cytokine-dependent thrombopoiesis in the bone marrow. However, ET with *CALR* mutations was not included in our previous study because *CALR* mutations have not yet been discovered in MPNs when we conducted our study in 2013. The discovery of *CALR* mutations in *JAK2*/*MPL*-unmutated ET patients in December 2013 have prompted us to ask the question that whether increased B cell activation can also be found in ET with *CALR* mutations similar to that in *JAK2*V617F-mutated ET [7–9]. Hence, we sought to screen for *CALR* mutations in a cohort of adult Taiwanese ET patients and to evaluate B cell immune profiles in *JAK2*V617F-mutated, *CALR*-mutated and triple-negative ET in this study.

## RESULTS

### Mutational analysis

Among 54 ET patients (median age at diagnosis 54.5 years; 54% females; median follow-up 4.4 years), 27 (50%) patients harbored the *JAK2*V617F mutation and one (1.9%) patient harbored the *MPL* W515K mutation. By nucleotide sequencing and HRMA, 19 (35.2% overall and 68.2% in *JAK2*/*MPL*-unmutated cases) patients harbored 8 types of *CALR* exon 9 mutations: 2 type 1 (p.L367fs*46), 10 type 2 (p.K385fs*47), 2 type 3 (p.L367fs*48), 1 type 34 (p.K385fs*47), and 4 other types (one each of p.L367fs*43, p.E370fs*60, p.E371fs*59 and p.E381del). Except p.E381del which is a 3 base-pair inframe deletion, all other *CALR* exon 9 mutations are indels causing +1 base-pair reading frameshift, with type 2 (10/19, 52.6%) being the most prevalent mutational type. One patient with *JAK2*V617F mutation harbored a single nucleotide polymorphism in *CALR* exon 9 (c.1142 A > C, rs143880510). Four (21%) of the 19 *CALR*-mutated patients had simultaneous *JAK2*V617F mutation; one each of type 3, p.E370fs*60, p.E371fs*59 and p.E381del, and the latter 3 *CALR* mutations were only detected by HRMA and required TA-cloning to confirm the presence of mutations indicating that they were low allelic burden mutants. Seven patients (13%) were triple-negative (TN) for *JAK2*, *CALR* and *MPL* mutations. No *DNMT3A* exon 23 or *IDH1*/*2* exon 4 mutation was detected in this cohort of ET patients. The only one *MPL*-mutated and the 4 *CALR*/*JAK2*V617F co-mutated ET patients were excluded from further clinical and molecular correlation analysis to avoid statistical bias.

### Clinical and molecular correlates

In 49 ET patients used for analysis, there was no significant difference in gender among the three major mutational groups. In this cohort, ET patients with *CALR* mutations had statistically significant longer follow-up (median 6.2 year, *p* = 0.031, Table 1), highest platelet count at the time of diagnosis (*p* = 0.01), and lower hemoglobin level at the time of diagnosis (*p* = 0.037). When compared with *JAK2*V617F-mutated ET patients, *CALR* mutations also correlated with younger age at diagnosis (*p* = 0.04) and lower leukocyte count (*p* = 0.013). *JAK2*V617F mutation was associated with leukocytosis (*p* = 0.002) and white blood cell count was lowest in TN ET patients.

**Table 1:** Clinical and laboratory characteristics in healthy adults and patients with essential thrombocythemia

Variables	HA (n=48)	*JAK2* mutation (n= 27)	*CALR* mutations (n=15)	Triple- negative (n=7)	*CALR* mutations vs *JAK2* mutation vs Triple- negative*p* value	*CALR* mutations vs *JAK2* mutation*p* value	*CALR* mutations vs Triple- negative*p* value	*JAK2* mutation vs Triple- negative*p* value	*CALR* mutations vs HA*p* value	*JAK2* mutation vs HA*p* value	Triple- negative vs HA*p* value
Male/Female gender, n (%)	15/33 (31/69)	11/16 (41/59)	7/8 (47/53)	3/4 (43/57)	NS	NS	NS	NS	-	-	-
Age at diagnosis (y), median (range)	-	55 (25-89)	45 (21-76)	52 (20-79)	NS	0.04	NS	NS	-	-	-
Follow-up (y), median (range)	-	3.6 (0.1-20.8)	6.2 (0.8-13.4)	3.3 (0.1- 10.3)	0.031	0.019	0.039	NS	-	-	-
Hemoglobin at diagnosis (g/dL), median (range)	-	13.5 (8.6-17.1)	11.9 (8.5-15.2)	12.9 (10.1-15.2)	0.037	0.013	NS	NS	-	-	-
WBC at diagnosis (x10^9^/L), median (range)	-	12.3 (5.7-27.7)	8.7 (4.3-17.5)	7.8 (5.3-10.2)	0.002	0.013	NS	0.002	-	-	-
Platelet at diagnosis (x10^9^/L), median (range)	-	948 (335-1437)	1275 (759-2606)	900 (608-1374)	0.01	0.004	0.039	NS	-	-	-
Hemoglobin at testing (g/dL), median (range)	12.9 (10.3-16.7)	13.4 (7.2-15.9)	12.5 (9.1-15)	13.3 (10.1-15.6)	NS	NS	NS	NS	NS	0.039	NS
WBC at testing (x10^9^/L), median (range), n=56	5.5 (3.9-7)	12.0 (6.8-21.8)	8.3 (4-13.8)	7.8 (4.6-8.6)	< 0.001	0.001	NS	0.001	0.005	< 0.001	0.034
Platelet at testing (x10^9^/L), median (range)	241.5 (118-366)	842 (449-1227)	734(247-2215)	824 (551-1127)	NS	NS	NS	NS	< 0.001	< 0.001	< 0.001
Cytoreductive therapy with hydroxyurea, n (%)	-	20 (74.1)	11 (73.3)	3 (42.9)	NS	NS	NS	NS	-	-	-

Abbreviations: HA, healthy adults; No. and n, number; NS, not significant; WBC, white blood cell; y, year.

### Distribution of B cells and B cell subsets

Among 49 ET patients in the three major mutational groups, there were no significant differences in the number of total B cells and all the B cell subset populations (Table 2). When compared to healthy adults, ET patients had significantly lower numbers of total B cells and naïve B cells, but had significantly higher number of plasmablast in all three mutational groups. The number of memory B cells was statistically lower in *CALR-* and *JAK2* mutated-ET patients when compared with healthy adults. There were no statistically significant differences in the numbers of early and late transitional B cells and pre-germinal center B cells between ET patients and healthy adults.

**Table 2:** Univariate analysis of B cell immune profiles in healthy adults and patients with essential thrombocythemia

Variables	HA (n=48)	*JAK2* mutation *(n= 27)*	*CALR* mutations (n=15)	Triple-negative (n=7)	*CALR* mutations vs *JAK2* mutation vs Triple-negativep value	*CALR* mutations vs *JAK2* mutationp value	*CALR* mutations vs Triple-negativep value	*JAK2* mutation vs Triple-negativep value	*CALR* mutations vs HAp value	*JAK2* mutation vs HAp value	Triple-negative vs HAp value
CD19+ B cells (/μL), median (range), n=71	230.5 (143-455)	129.0 (25.8-358)	121.8 (17.8-318)	97.2 (38.1-219.5)	NS	NS	NS	NS	0.001	< 0.001	0.001
Early transitional B cells (T1) (/μL), median (range), n=61	3 (1-11)	1.1 (0.0-15.5)	2 (0.0-19.4)	2.5 (1-10)	NS	NS	NS	NS	NS	NS	NS
Late transitional B cells (T2) (/μL), median (range), n=61	7 (3-15)	15 (1-46)	14 (0.4-29.5)	9.2 (2-46)	NS	NS	NS	NS	NS	NS	NS
Pre-germinal center B cells (/μL), median (range), n=61	4 (1-24)	7 (2-17.6)	6.6 (2-19)	4.0 (3-25)	NS	NS	NS	NS	NS	NS	NS
Memory B cells (/μL), median (range), n=61	75.5 (32-181)	34 (6.8-108)	34 (3.4-145)	28.5 (20-165)	NS	NS	NS	NS	< 0.001	< 0.001	NS
Plasmablast (/μL), median (range), n=61	0.0 (0.0-1)	0.4 (0.0-4)	0.3 (0.0-3)	0.5 (0.0-2)	NS	NS	NS	NS	< 0.001	< 0.001	0.025
Naive B cells (/μL), median (range), n=61	134.5 (75-238)	65.6 (5.8-293)	45.8(5.8-223)	57.5 (16-591)	NS	NS	NS	NS	0.003	0.01	0.014
MFI of mBAFF on granulocytes, n=63	6.7 (4.6-8.3)	25.4 (7.8-75.2)	34.2 (10.2-67.7)	33.7 (4.5-65.4)	NS	NS	NS	NS	< 0.001	< 0.001	0.007
MFI of mBAFF on monocytes, n=62	7.6 (2.8-8.0)	14.2 (3.5-54.6)	28.9 (4.4-49.4)	14.3 (3.2-40.7)	NS	NS	NS	NS	< 0.001	< 0.001	0.005
Serum BAFF level (ng/mL), n=66	1.1 (0.6-2.4)	2.3 (0.8-4.9)	1.6 (0.9-3.9)	1.8 (1.4-3.8)	0.049	0.02	NS	NS	0.015	< 0.001	0.001
IL-6 in B cells (%), n=39	1.0 (0.2-1.9)	6.7 (2.6-9.6)	6.8 (4.0-13.7)	8.2 (4.5-10.3)	NS	NS	NS	NS	< 0.001	< 0.001	< 0.001
IL-1β in B cells (%), n=39	4.8 (0.9-13.4)	11.5 (1.4-32.5)	15.4 (4.6-32.6)	12.9 (3.6-41.7)	NS	NS	NS	NS	0.002	0.002	0.012
TLR4 in B cells (%), n=54	2.3 (0.3-3.0)	4.5 (0.4-24.2)	11.3 (2.3-22.8)	3.4 (1.0-5.4)	0.021	NS	0.001	NS	< 0.001	0.001	0.02
CD69+ B cells (/μL), median (range), n=48	2.2 (1.0-4.6)	12.5 (0.6-39.1)	20.8 (2.5-51.4)	7.6 (1.8-18.6)	NS	NS	0.048	NS	< 0.001	0.002	NS
CD80+ B cells (/μL), median (range), n=46	9.2 (0.8-12.5)	9.0 (1.9-79.1)	13.8 (1.0-72.6)	10.0 (1.6-44.7)	NS	NS	NS	NS	0.036	NS	NS
CD86+ B cells (/μL), median (range), n=46	10.9 (3.0-41.9)	26.3 (4.3-101.6)	18.3 (5.9-63.4)	24.2 (8.0-82.3)	NS	NS	NS	NS	0.041	0.012	NS

Abbreviations: BAFF, B cell-activating factor; HA, healthy adults; IL, interleukin; mBAFF, membrane-bound B cell-activating factor; MFI, mean fluorescence intensity; No. and n, number; NS, not significant; TLR4, toll-like receptor 4; WBC, white blood cell; y, year.

### B cell immune profiles

**Figure 1:**
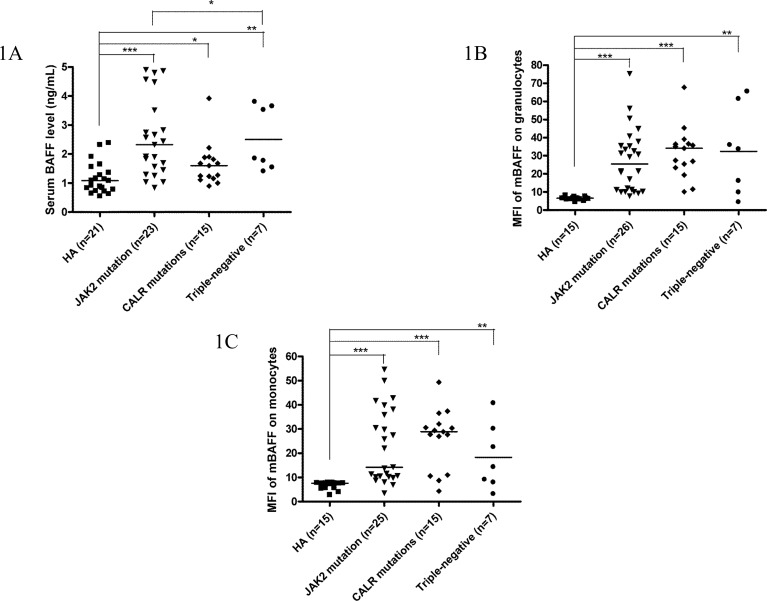
Elevated serum BAFF levels and higher membrane-bound BAFF expression in peripheral granulocytes and monocytes of ET patients **1A**, Elevated serum BAFF levels were found in ET patients, regardless of genotypes compared to healthy adults. *CALR*-mutated ET patients had lowest serum BAFF levels compared to *JAK2*-mutated and triple-negative ET patients in univariate analysis. **1B** and **1C**, Membrane-bound BAFF expression in peripheral granulocytes and monocytes was higher in ET patients, regardless of genotypes compared to healthy adults, respectively. Median values are indicated by the short horizontal bars. Asterisks represent significant differences between groups. *p < 0.05, **p < 0.01, ***p < 0.001.

**Figure 2:**
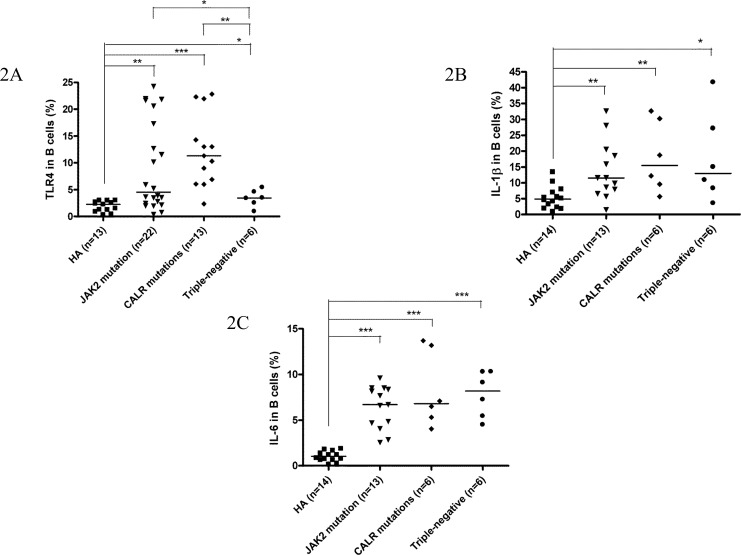
Fractions of activated B cells with TLR4 expression, and IL-1β and IL-6 production were higher in ET patients **2A**, B cells from patients with ET, regardless of genotypes expressed significantly higher levels of TLR4 compared to healthy adults. *CALR*-mutated ET patients had highest TLR4 expression compared to *JAK2*-mutated and triple-negative ET patients. **2B** and **2C**, B cells from patients with ET, regardless of genotypes expressed significantly higher levels of IL-1β and IL-6 compared to healthy adults, respectively. Median values are indicated by the short horizontal bars. Asterisks represent significant differences between groups. *p < 0.05, **p < 0.01, ***p < 0.001.

**Figure 3:**
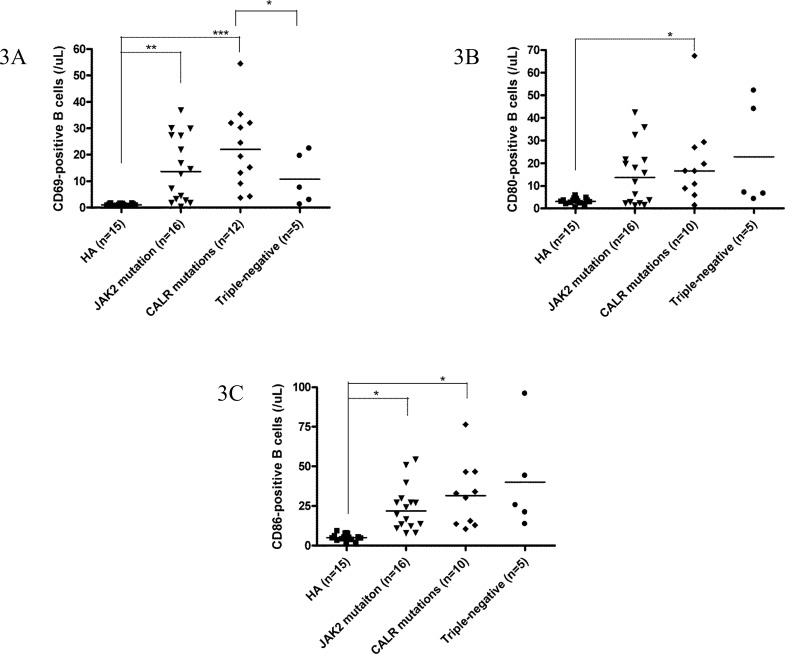
***CALR*** mutations were associated with activated B cells in patients with ET. **3A, 3B** and **3C**, The number of activated B cells was higher in *CALR*-mutated ET patients, as evidenced by expression of CD69, CD80 and CD86, respectively. Data are presented as the number of B cells expressing these markers. Median values are indicated by the short horizontal bars. Asterisks represent significant differences between groups. *p < 0.05, **p < 0.01, ***p < 0.001.

Among 49 ET patients in the three major mutational groups, the B cell immune profiles in 34 (69.4%; 19 *JAK2*V617F-mutated, 9 *CALR*-mutated and 6 TN) patients had been previously described [23]. When compared with *JAK2*V617F-mutated and TN ET patients, *CALR* mutations correlated with significantly lower serum BAFF level (median 1.6 ng/mL, *p* = 0.049) (Figure 1A) and higher fraction of B cells with TLR4 expression (median 11.3%, *p* = 0.021) (Figure 2A). Besides, ET patients with *CALR* mutations had statistically higher number of CD69-positive activated B cells when compared to TN group (median: 20.8/μL *vs* 7.6/μL, *p* = 0.048) (Figure 3A). There were no significant differences in mean fluorescence intensity (MFI) of mBAFF on both granulocytes and monocytes (Figure 1B and 1C, respectively), in the fraction of B cells with intracellular IL-1β or IL-6 expression (Figure 2B and 2C, respectively), and the numbers of CD80-positive and CD86-positive activated B cells among the three mutational groups of ET patients (Figure 3B and 3C, respectively).

When compared to healthy adults, patients with ET had statistically significant higher serum BAFF level and higher MFI of mBAFF on both granulocytes and monocytes (Figure 1), and higher fraction of B cells with TLR4 expression and higher fractions of B cells with intracellular IL-1β and IL-6 expression irrespective of their genotypes (Figure 2) (Table 2). Although ET patients had significantly lower numbers of CD19-positive B cells and naïve B cells when compared to healthy adults, ET patients with *CALR* and *JAK2* mutations had statistically higher numbers of CD69-positive and CD86-positive activated B cells (Figure 3A and 3C, respectively). 38 (70.4%) of 54 ET patients were treated with hydroxyurea to lower their blood counts in this cohort. There were no significant differences in all the B cells immune profiles in ET patients with or without hydroxyurea treatment, except lower IL-1β expression level in B cells (median 6.9% *vs* 16.4%, *p* = 0.014) was found in ET patients being treated with hydroxyurea (Table 1 and Supplementary Table S1).

In this study, platelet count at testing had moderately positive correlation with the fractions of B cells with intracellular IL-1β and IL-6 expression (Table 3). MFI of mBAFF on granulocytes had strong positive correlation with MFI of mBAFF on monocytes, and had moderately positive correlation with the fractions of B cells with intracellular IL-1β and IL-6 expression. In addition, serum BAFF levels had moderately positive correlation with the fraction of B cells with intracellular IL-6 expression. Interestingly, only MFI of mBAFF on granulocytes, but not MFI of mBAFF on monocytes or the serum BAFF levels, had weak positive correlation with the numbers of CD69-positive and CD86-positive activated B cells in our cohort. We also analyzed the correlation between platelet count, serum BAFF levels, and B cell immune profiles in the group of healthy controls. Platelet count of healthy controls only had moderately negative correlation with the MFI of mBAFF on monocytes (Spearman's rho = -0.625, *p* = 0.013) suggesting that the platelet count of healthy controls did not have obvious correlation with their B cell immune profiles.

**Table 3:** Correlation of platelet count at testing, serum BAFF levels, and B cell immune profiles in this study

Variables	Platelet at testing		MFI of mBAFF on granulocytes		MFI of mBAFF on monocytes		Serum BAFF levels	
	Spearman's rho	*p* value	Spearman's rho	*p* value	Spearman's rho	*p* value	Spearman's rho	*p* value
MFI of mBAFF on granulocytes	0.377	0.002	-	-	0.786	<0.001	0.29	0.021
MFI of mBAFF on monocytes	0.312	0.011	0.786	<0.001	-	-	0.304	0.016
Serum BAFF levels	0.486	<0.001	0.29	0.021	0.304	0.016	-	-
IL-6 in B cells	0.61	<0.001	0.545	<0.001	0.398	0.009	0.572	<0.001
IL-1β in B cells	0.543	<0.001	0.55	<0.001	0.472	0.002	0.293	NS
CD69+ B cells	0.325	0.021	0.388	0.005	0.161	NS	0.225	NS
CD86+ B cells	0.26	NS	0.42	0.003	0.252	NS	0.141	NS

Abbreviations: BAFF, B cell-activating factor; IL, interleukin; mBAFF, membrane-bound B cell-activating factor; MFI, mean fluorescence intensity; NS, not significant.

### Multivariate analysis of B cell immune profiles in ET

The results of multivariate analysis using linear regression model adjusted for multiple parameters confirmed that increased activated CD69+ B cells were universally present in *JAK2*-mutated, *CALR*-mutated and triple-negative ET patients when compared to healthy adults, although the number of total B- cells was significantly lower in ET patients (Table 4). Activated B cells were characterized by the expression of CD69 and CD86, increased intracellular IL-6 and IL-1β levels, and higher expression of TLR4. Interestingly, peripheral granulocytes and monocytes mBAFF expression was significantly higher in ET patients compared to healthy controls. *JAK2*-mutated and *CALR*-mutated ET patients had significantly higher number of B-cells expressing TLR4 and IL-6, and TN ET patients had significantly higher number of B-cells expressing IL-6 and IL-1β (Table 4). TN ET patients had significantly lower number of B-cells expressing TLR4 when compared to *CALR*-mutated and *JAK2*-mutated ET patients (Table 5). TN ET patients also had significantly lower number of CD69+ B-cells when compared to *CALR*-mutated ET patients.

**Table 4:** Comparison of B cell immune profiles between healthy adults and patients with essential thrombocythemia using linear regression model adjusted for hematological parameters

Variables	ET subgroups	Unstandardized Coefficients		*p* value	95% Confidence Interval for B	
		B	Std. Error		Lower Bound	Upper Bound
CD19+ B cells (/μL)	*JAK2*-mutated	−109.918	42.713	0.013	−195.846	−23.990
	*CALR*-mutated	−102.613	40.102	0.014	−183.289	−21.938
	TN	−148.166	46.096	0.002	−240.899	−55.433
Neutrophil mBAFF MFI	*JAK2*-mutated	25.969	9.115	0.006	7.642	44.297
	*CALR*-mutated	33.242	8.565	<0.001	16.020	50.463
	TN	34.018	9.410	0.001	15.099	52.938
Monocyte mBAFF MFI	*JAK2*-mutated	14.897	7.287	0.047	0.237	29.556
	*CALR*-mutated	23.159	6.779	0.001	9.522	36.796
	TN	16.591	7.446	0.031	1.612	31.571
Serum BAFF levels (ng/ml)	*JAK2*-mutated	0.915	0.625	NS	−0.343	2.173
	*CALR*-mutated	0.194	0.586	NS	−0.986	1.373
	TN	1.102	0.643	NS	−0.194	2.397
Total B IL-6 %	*JAK2*-mutated	4.488	1.912	0.027	0.551	8.425
	*CALR*-mutated	4.906	2.337	0.046	0.093	9.719
	TN	5.463	1.724	0.004	1.913	9.014
Total B IL-1β%	*JAK2*-mutated	11.566	7.807	NS	−4.513	27.644
	*CALR*-mutated	16.529	9.544	NS	−3.128	36.186
	TN	14.697	7.040	0.047	0.199	29.196
Total B TLR4 %	*JAK2*-mutated	14.090	4.281	0.002	5.439	22.742
	*CALR*-mutated	14.262	3.746	<0.001	6.692	21.832
	TN	5.888	4.378	NS	−2.961	14.737
CD69+ B cells (/μL)	*JAK2*-mutated	35.629	9.431	0.001	16.440	54.817
	*CALR*-mutated	34.581	7.583	<0.001	19.153	50.008
	TN	19.206	9.302	0.047	0.282	38.130
CD80+ B cells (/μL)	*JAK2*-mutated	18.875	15.117	NS	−11.956	49.705
	*CALR*-mutated	15.519	12.409	NS	−9.790	40.828
	TN	16.876	14.963	NS	−13.640	47.393
CD86+ B cells (/μL)	*JAK2*-mutated	38.875	17.586	0.035	3.007	74.742
	*CALR*-mutated	27.556	14.437	NS	−1.888	57.000
	TN	33.042	17.407	NS	−2.460	68.545

Abbreviations: mBAFF, membrane-bound B cell-activating factor; IL: interleukin; MFI, mean fluorescence intensity; NS, not significant; Std., standard; TLR4: toll-like receptor 4; TN, triple-negative.

**Table 5:** Comparison of B cell immune profiles among patients with essential thrombocythemia using linear regression model adjusted for age, sex, follow-up period and hematological parameters

Variables	ET subgroups	*CALR*-mutated group as control					*JAK2*-mutated group as control				
		Unstandardized Coefficients		*p* value	95% Confidence Interval for B		Unstandardized Coefficients		*p* value	95% Confidence Interval for B	
		B	Std. Error		Lower Bound	Upper Bound	B	Sth. Error		Lower Bound	Upper Bound
Serum BAFF levels (ng/ml)	*JAK2*-mutated	0.436	0.479	NS	−0.536	1.408	−	−	−	−	−
	*CALR*-mutated	−	−	−	−	−	−0.436	0.479	NS	−1.408	0.536
	TN	0.571	0.578	NS	−0.601	1.743	0.135	0.577	NS	−1.035	1.304
Total B TLR4 %	*JAK2*-mutated	0.437	2.759	NS	−5.183	6.057	−	−	−	−	−
	*CALR*-mutated	−	−	−	−	−	−0.437	2.759	NS	−6.057	5.183
	TN	−8.169	3.329	0.02	−14.951	−1.388	−8.606	3.467	0.019	−15.669	−1.543
CD69+ B cells (/μL)	*JAK2*-mutated	−1.303	7.025	NS	−15.801	13.196	−	−	−	−	−
	*CALR*-mutated	−	−	−	−	−	1.303	7.025	NS	−13.196	15.801
	TN	−18.480	8.265	0.035	−35.539	−1.421	−17.177	8.605	NS	−34.936	0.582
CD80+ B cells (/μL)	*JAK2*-mutated	6.135	11.382	NS	−17.470	29.740	−	−	−	−	−
	*CALR*-mutated	−	−	−	−	−	−6.135	11.382	NS	−29.740	17.470
	TN	3.273	13.175	NS	−24.051	30.597	−2.862	13.175	NS	−30.185	24.462
CD86+ B cells (/μL)	*JAK2*-mutated	16.243	13.696	NS	−12.161	44.647	−	−	−	−	−
	*CALR*-mutated	−	−	−	−	−	−16.243	13.696	NS	−44.647	12.161
	TN	11.584	15.854	NS	−21.294	44.463	−4.658	15.854	NS	−37.537	28.221

Abbreviations: BAFF, B cell-activating factor; NS, not significant; Std., standard; TLR4: toll-like receptor 4; TN, triple-negative.

**Table 6:** Characteristics and the frequency of CALR and JAK2V617F co-mutations in patients with essential thrombocythemia

Author	Population	Method to detect *CALR* mutations	*CALR* mutations	Frequency of *CALR* and *JAK2*V617F co-mutations		
				In ET no. (%)	In *CALR*-mutated ET no. (%)	In *JAK2*V617F-mutated ET no. (%)
Lundberg *et al*. [28]	Caucasian	Allele-specific PCR	p.K385fs*47	1/69 (1.4)	1/17 (5.9)	1/41 (2.4)
Fu *et al*. [29]	Chinese	Sanger sequencing	L367fs*46 c.997 C>T (arginine>tryptophan)	2/436 (0.5)	2/99 (2)	2/240 (0.8)
Shirane *et al*. [30]	Japanese	Fragment analysis and deep sequencing	p.E378fs*45	1/111 (0.9)	1/22 (4.5)	1/60 (1.7)
Ha and Kim [31]	Korean	Sanger sequencing	p.L367fs*46	1/114 (0.9)	1/25 (4)	1/68 (1.5)
Al Assaf *et al*.[32]	Caucasian	Sanger sequencing	p.K385fs*47	1/160 (0.6)	1/59 (1.7)	1/57 (1.8)
Lin *et al*.[33]	Chinese	Sanger sequencing	2 p.L367fs*462 p.K385fs*47	4/428 (0.9)	4/101 (4.0)	4/254 (1.6)
Lim *et al*. [34]	Taiwanese	HRMA andSanger sequencing	p.L367fs*48 p.E381A p.K385fs*47 p.E370fs*60 p.E371fs*59 p.E371del p.E378del p.E396del p.E374X p.E380X p.K391X p.E372G p.E380G	13/92 (14.1)	13/34 (38)	13/59 (22)
Usseglio *et al*. [35]	Caucasian	HRMA	2 p.K385fs*47 p.L367fs*48 c.1125_1147del	4/103 (3.9)	4/48 (8.3)	4/56 (7.1)
Lim *et al*. (this study)	Taiwanese	HRMA andSanger sequencing	p.L367fs*48 p.E370fs*60 p.E371fs*59 p.E381del	4/54 (7.4)	4/19 (21.1)	1/31 (3.2)

Abbreviations: ET, essential thrombocythemia; HRMA, high-resolution melting analysis; no., number.

## DISCUSSION

*CALR* mutations have been found to have phenotypic and prognostic significances in patients with ET from both Caucasian and Chinese populations [7, 8, 11, 24–27]. In this cohort of adult Taiwanese ET patients, *CALR* mutations were found to have a similar phenotypic correlation with higher platelet count, lower hemoglobin level and younger age at diagnosis. However, we detected a higher frequency of type 2 *CALR* mutation (10 of 19 patients) in this study while there was only 2 type 1 *CALR* mutation detected. These results are contradictory to the vast majority of the reports in the literature. The possible explanations for this discrepancy in our results might be related to small sample size and selection bias cannot be excluded completely in this study.

In accordance with our previous report, a relatively high frequency of *CALR* and *JAK2*V617F co-mutations (21% in 19 *CALR*-mutated ET) was still found in this study. Several papers have reported the co-occurrence of *CALR* and *JAK2*V617F mutations in ET across different ethnic groups including one of our previous publication (Table 6). The frequency of *CALR* and *JAK2*V617F co-mutations ranges from 0.5 to 14.1%, 1.7 to 38%, and 0.8 to 22%, in ET, *CALR*-mutated ET, and *JAK2*V617F-mutated ET, respectively [28–35]. The cause of the difference in the frequency of *CALR* and *JAK2*V617F co-mutations in these studies might be related to the different methods used to detect *CALR* mutations. Higher frequency of *CALR* and *JAK2*V617F co-mutations was detected by using HRMA, whereas Sanger sequencing will likely miss to detect low allelic burden (< 10%) *CALR* mutants. On the other hand, Usseglio *et al*. found that *CALR* mutations could be detected in low allelic burden (< 4%) *JAK2*V617F-mutated ET suggesting that the frequency of *CALR* and *JAK2*V617F co-mutations might be further increased if a highly sensitive test was employed to detect *JAK2*V617F mutation in *CALR*-mutated ET [35]. Since both of our studies used a sensitive in-house developed HRMA followed by TA-cloning to detected *CALR* mutations, we were able to identify many low allelic burden *CALR* mutants resulting in the higher frequency of *CALR* and *JAK2*V617F co-mutations in our series. However, because our study was limited by small patient size, larger study using sensitive screening methods for the detection of both *CALR* and *JAK2*V617F mutations will be warranted to confirm our results.

Recently, we have shown that ET patients have quantitative and qualitative changes in their B cell immune profiles regardless of *JAK2*V617F mutational status [23]. In our previous report, we found that the number of CD19+ B cells did not differ between ET patients and age-matched healthy adults using univariate analysis. However, we found that ET patients had significantly lower numbers of total CD19+ B cells in univariate analysis (Table 2) and also in multivariate analysis adjusted for age, sex, follow-up period and hematological parameters (Table 4) in this study. We believe that the results reported in this study are more accurate because *CALR*-mutated ET patients were not included in our previous report and the results from multivariate analysis are more reliable. In the present study, we found that ET patients with *CALR* mutations also had similar quantitative and qualitative changes in most of the B cell immune profiles when compared to healthy adults using univariate and multivariate analyses (Tables 2 and 4, respectively). Although the number of total B cells was lower in ET patients including those with *CALR* mutations when compared with healthy controls, the number of activated B cells was significantly increased in ET patients across all 3 genotypes that characterized by the expression of CD69 and CD86, increased intracellular IL-6 and IL-1β levels, and higher expression of TLR4.

Regarding to the mechanism of B cell activation in ET patients, it has been well documented that elevated serum levels of inflammatory cytokines are frequently detected in patients of MPN, especially PMF, and may correlate to their constitutional symptoms which could be effectively ameliorated by the use of JAK inhibitor [36]. Previous study has reported that cytokine levels were also significantly increased in ET and PV patients [37]. Therefore, it is reasonable to argue that B cell activation could only be an epiphenomenon in ET rather than a cause of thrombopoiesis. However, we found that increased B cell activation was only present in ET patients but not in PV patients when compared to healthy controls or patients with reactive thrombocytosis (Supplementary Table S2). Although we did not evaluate B cell immune profiles in PMF patients due to difficulty in patient enrollment, our findings provided evidence to illustrate that increased B cell activation in ET patients could not be solely explained by the increased cytokine levels in MPN patients, and therefore might not be an epiphenomenon in these patients. Nevertheless, we had previously reported that some humoral factors such as endogenous toll-like receptor 4 (TLR4) ligands HSP70 and HMGB1 or other inflammatory cytokines, might participate in the activation of B cells in ET patients because peripheral B cells of ET patients could be stimulated by ET patients’ sera to cause IL-1beta and IL-6 production [23]. In addition, we had also demonstrated that increased production of BAFF by granulocytes and monocytes up-regulates TLR4 expression on B cells and promotes B cell activation in ET patients. Consequently, these activated B cells play a pathogenic role in augmenting thrombocytosis by producing IL-1β and IL-6 in ET patients through cytokine-dependent thrombopoiesis in the bone marrow. Altogether, our data suggested that increased B cell activation in ET might be caused by the stimulation of specific humoral factors on B cells and the interaction of B cells with BAFF on granulocytes and monocytes. Importantly, our studies suggested that activated B cells in ET could play a role in mediating pathogenic thrombopoiesis in the bone marrow.

Besides, we had previously reported that TLR4 expression is upregulated in both naïve and memory B cell subsets, and BAFF receptor signaling has reciprocal effects on TLR interaction [23]. B cells are characterized by the expression of a clonally rearranged, antigen-specific B cell receptor (BCR) in combination with the expression of one or more members of the TLRs [38]. This dual expression feature allows B cells to integrate both antigen-specific signals and environmental danger signals *via* these key receptor systems. Since we did not measure or characterize the expression level of BCR on B cells, whether dual BCR and TLRs engagement may also play a role in the activation and/or affect the function of B cells in ET patients remains to be elucidated in future study.

Furthermore, we did not favor the paracrine effect of serum BAFF secreted by peripheral granulocytes and monocytes because its level was not different between ET patients and healthy controls in multivariate analysis. Rather, we hypothesized that the direct interaction between peripheral granulocytes and monocytes and B-cells might play a role in the activation of B-cells in ET patients since mBAFF expression was significantly higher in ET patients compared to healthy controls. Recently, mBAFF has been found to be a more potent stimulus for B cells than soluble BAFF thus supporting our view [39]. Our observation was also supported by the finding that mBAFF expression on peripheral granulocytes significantly correlated with higher number of IL-1β/IL-6-producing B cells and activated B-cells in ET patients (Table 3). In addition, higher number of TLR4-producing B cells in *JAK2*-mutated and *CALR*-mutated ET patients might also augment the production of IL-1β/IL-6 in B cells in these patients. We had previously shown that IL-1β and IL-6 play an important role in thrombopoiesis in ET patients, and hematopoietic stem cells of ET patients differentiated towards a megakaryocytic lineage after incubation with their own B cells [23]. Therefore, our data suggested that activated B-cells in ET patients might link to the pathogenic thrombopoiesis in these patients through the production of IL-1β/IL-6 in activated B cells regardless of their genotypes.

It is possible that the use cytoreductive therapy might affect B cell immune profiles in ET patients. However, most B cell immune profiles in ET patients were not affected by the treatment of hydroxyurea in this study (Supplementary Table S1). Therefore, we believed that the changes in B cell immune profiles may be more closely related to the underlying pathogenic mechanisms that could not be altered by non-specific cytoreductive therapy such as hydroxyurea.

Currently, the exact molecular mechanism of B cell activation in ET patients has not yet been fully elucidated. However, most of the changes in B cell immune profiles are independent of the three genotypes in ET patients, and the activation of JAK-STAT signaling pathway can be seen in most ET patients regardless of their molecular profiles [40]. *JAK2*V617F is a gain-of-function mutation resulting in the cytokine-independent growth of hematopoietic progenitors [41]. However, *JAK2*V617F mutation requires the presence of cytokine receptors (especially MPL) to be constitutively active [42]. *JAK2*V617F mutation can activate erythropoietin receptor, thrombopoietin receptor or granulocyte colony-stimulating factor receptor on progenitor cells to promote erythropoiesis, megakaryopoiesis, or granulopoiesis. Interestingly, *CALR* mutations are recently found to activate the JAK-STAT signaling through a MPL-dependent mechanism, and cause thrombocytosis both *in vitro* and *in vivo*. Hence, both *JAK2*V617F and *CALR* mutations can activate the JAK-STAT signaling in megakaryocytes. Although *CALR* mutations can be detected in hematopoietic stem/progenitor cells, it largely promotes the growth and the differentiation of megakaryocytic precursors resulting in the phenotype of ET and/or PMF. Therefore, *CALR* mutations are exclusively detected in around 25 % of ET or PMF, but not in PV. On the other hand, *JAK2*V617F mutation can be identified in about 95% of PV and in around 60 % of ET or PMF. Several observations have suggested that megakaryocytes play a major role in the pathogenesis of MPNs [43]. There is evidence suggesting that MPN associated mutations could alter megakaryocyte differentiation, migratory ability, and proplatelet formation, leading to increased platelet production [44]. *JAK2*V617F mutation was also found to lead to intrinsic changes in both megakaryocyte and platelet biology in a mouse model of ET [45]. Recently, CALR mutations have been shown to activate essential MAPK signaling through MPL-dependent mechanism and facilitate megakaryocyte differentiation [46]. Current evidences suggest that both *JAK2*V617F and *CALR* mutations intrinsically play a major role in the pathogenesis of ET through the promotion of megakaryopoiesis and thrombopoiesis. Based on our findings, increased platelet production in ET patients may be resulted from activating mutations synergistic with bystander thrombopoietic cytokines produced by activated B cells. We believe that these results would help advance our understanding of the pathogenesis of ET.

Our study is limited by a total number of 54 ET patients. However, the distribution and the percentage of the 3 driver mutations in these 54 ET patients were comparable with most studies: 27 (50%) patients harbored the *JAK2*V617F mutation, 1 (1.9%) patient with the *MPL*W515K mutation, 19 (35.2% overall and 68.2% in *JAK2*/*MPL*-unmutated cases) patients with *CALR* exon 9 mutations, and 7 (13%) TN patients. In this study, we detected a higher percentage of *CALR*/*JAK2*V617F co-mutations in 4 (7.4%) ET patients due to the use of a sensitive HRMA followed by TA-cloning to detect low allelic burden *CALR* mutants. To avoid statistic bias on the results, we excluded these 4 *CALR*/*JAK2*V617F co-mutated ET patients and the only one *MPL*-mutated ET patient from further analysis. We have also consulted our bio-statistician for help with the analysis of our data. Our results showed that increased B cell activation is present in *JAK2*V617F-mutated, *CALR*-mutated and triple-negative ET, and these findings are consistent with our previous report. Although we believe that there was no statistical bias on the results, larger study is still warranted to confirm our findings. In conclusion, increased B cell activation is present in ET patients across different mutational subgroups.

## PATIENTS AND METHODS

### Patient enrollment

The screening for mutations in patients with hematologic neoplasms was approved by the Institutional Review Board of MacKay Memorial Hospital (09MMHIS157 and 12MMHIS034). 54 adult Taiwanese ET patients were enrolled and written informed consent was obtained. The clinical and laboratory characteristics at the time of diagnosis/referral and at testing were determined retrospectively by chart review. Parts of the clinical data of 48 patients in this cohort have been described in our recent publication [10].

### Mutation screening

Genomic DNA derived from bone marrow granulocytes, peripheral blood leukocytes, peripheral blood granulocytes or peripheral blood mononuclear cells were used for the screening of *CALR* exon 9 mutations spanning codons 352-417 [GenBank: NM_004343]. Oligonucleotide primers targeting *CALR* exon 9 were used to amplify a 285 bp product: (*CALR* Forward 5′-CCTGCAGGCAGCAGAGAAAC-3′) (*CALR* Reverse 5′-ACAGAGACATTATTTGGCGCG-3′). The PCR were amplified using GoTaq Green Master Mix (Promega, CA, USA) on a Thermal Cycler^®^ PCR System 2720 (Applied Biosystems, CA, USA). The final concentrations were as follows: 3 mM MgCl2 and 0.4 mM deoxyribo-nucleotide triphosphate, 2.5 μM each of forward and reverse oligo primer, 50 ng of DNA template and water to a final reaction volume of 20μl. Cycling parameters consisted of an initial denaturation at 94°C for 5 min; 35 cycles of denaturation at 94°C for 30 s, annealing at 58°C for 30s, and extension at 72°C for 45s; and final extension at 72°C for 10 min. The EXO-SAP reagent (USB, CA, USA) was used to clean up the PCR product prior to sequencing. Direct DNA sequencing was conducted using the same primers for amplification and a BigDye terminator v3.1 Cycle sequencing kit (Applied Biosystems, CA, USA) on an ABI 3730 sequencer. Mutations were identified using DNA Dynamo sequence analysis software (Blue Tractor Software Ltd, Conwy, UK). All identified sequence variants were subjected to repeated bidirectional sequencing for confirmation. *CALR* exon 9 mutations were also independently screened by high-resolution melting analysis (HRMA) and TA-cloning was used to detected low allelic burden mutants in selected samples as previously described [47]. *JAK2*V617F mutation was determined by allele-specific PCR as previously described and/or mutation-enrich high sensitive PCR method over *JAK2* exon 14 mutation hot spot area [48, 49]. *MPL* exon 10 mutation was screened by nucleotide sequencing as previously described [50]. In order to exclude the influence of other possible mutations on B cell immune profiles, *DNMT3A* exon 23 and *IDH1*/*2* exon 4 mutations were also screened as previously described [50].

### B cell immune profiles

The quantification of B cell populations and various B cell subsets including T1, T2, pre-germinal center, memory, and plasmablast/plasma cells, based upon the surface expression of CD19, CD24, CD27, CD38, and IgD was assessed by flow cytometric analysis as previously described [23]. Granulocytes and monocytes membrane-bound BAFF (mBAFF) levels, TLR4 expression and intracellular levels of IL-1β/IL-6 and the expression of CD69, CD80, and CD86 on B cells were quantified by flow cytometry using appropriated antibodies [23]. Serum BAFF concentration was measured by ELISA kit from R&D Systems according to the manufacturer's instructions. The B cell immune profiles of 38 patients in this cohort had been described in our previous publication [23]. B cell immune profiles from 48 healthy adults were used for comparison.

### Statistical analysis

The correlation between *CALR* mutational status and clinical characteristics was calculated by the chi-square test or Fisher's exact test. Kolmogorov-Smirnov test was used to test normality of numerical variables. The independent *t*-test and the one-way analysis of variance (ANOVA) were used to compare differences between two and three independent groups when the dependent variables were normally distributed, respectively. When the dependent variables were not normally distributed, non-parametric Mann-Whitney U test and Kruskal-Wallis H test were used to compare differences between two and three independent groups, respectively. Spearman's rank correlation coefficient was used to evaluate the relationship between two variables. Multivariate analysis was performed using linear regression model adjusted for age, sex, follow-up period and hematological parameters. Statistical significance was defined as a two-sided *p* value < 0.05 and SPSS version 22.0 (IBM, New York, USA) was used for analyses.

## SUPPLEMENTARY MATERIALS TABLES


