# Supplementary material for: Increased B cell activation is present in JAK2V617F-mutated, CALR-mutated and triple-negative essential thrombocythemia
Source: Oncotarget. 2017 Mar 18;8(20):32476–91. doi: 10.18632/oncotarget.16381 (PMC5464803; doi:10.18632/oncotarget.16381)
Supplement: Supplementary file 1 [file oncotarget-08-32476-s001.pdf]

Increased B cell activation is present in *JAK2*V617F-mutated, *CALR*-mutated and triple-negative essential thrombocythemia

Supplementary Material

Supplementary Table 1: Laboratory characteristics and B cell immune profiles in essential thrombocythemia patients with and without treatment with hydroxyurea.

Variables	No	Yes	p value
Cytoreductive therapy with hydroxyurea, n (%)	16 (29.6)	38 (70.4)	-
Hemoglobin at testing (g/dL), median (range), n=54	13.4 (9.1-17.7)	13.4 (7.2-15.8)	NS
WBC at testing (x10^9/L), median (range), n=54	8.2 (4.0-29.3)	10.5 (4.4-19.7)	NS
Platelet at testing (x10^9/L), median (range), n=54	850.0 (333.0-1369.0)	728.5 (247.0-2215.0)	NS
CD19+ B cells (/µL), median (range), n=52	139.0 (17.8-358.0)	121.8 (25.8-2031.9)	NS
Early transitional B cells (T1) (/µL), median (range), n=37	2.0 (0.1-10.0)	2.5 (0.0-19.4)	NS
Late transitional B cells (T2) (/µL), median (range), n=37	11.4 (0.4-46.0)	15.0 (1.0-46.0)	NS
Pre-germinal center B cells (/µL), median (range), n=37	6.9 (2.4-25.0)	6.8 (2.0-19.0)	NS
Memory B cells (/µL), median (range), n=37	21.2 (6.0-165.0)	35.1 (3.4-145.0)	NS
Plasmablast (/µL), median (range), n=37	0.3 (0.0-2.0)	0.4 (0.0-4.0)	NS
Naive B cells (/µL), median (range), n=37	48.2 (5.8-591.0)	59.0 (5.8-367.0)	NS
MFI of mBAFF on granulocytes, n=52	35.3 (7.8-65.4)	23.5 (4.5-75.2)	NS
MFI of mBAFF on monocytes, n=51	27.7 (8.0-38.1)	18.1 (3.2-54.6)	NS
Serum BAFF level (ng/mL), n=49	1.7 (0.8-4.8)	1.9 (0.9-4.9)	NS
IL-6 in B cells (%), n=28	9.4 (2.7-13.6)	8.1 (2.8-13.6)	NS
IL-1β in B cells (%), n=28	16.4 (5.6-32.1)	6.9 (4.1-17.9)	0.014
TLR4 in B cells (%), n=44	22.3 (13.1-103.3)	19.1 (6.9-134.4)	NS
CD69+ B cells (/µL), median (range), n=35	14.2 (2.5-35.8)	13.2 (0.5-943.4)	NS
CD80+ B cells (/µL), median (range), n=33	26.3 (3.0-72.6)	13.8 (1.0-449.7)	NS
CD86+ B cells (/µL), median (range), n=33	21.7 (5.9-82.3)	26.8 (4.3-191.7)	NS

Abbreviations: BAFF, B cell-activating factor; IL, interleukin; mBAFF, membrane-bound B cell-activating factor; MFI, mean fluorescence intensity; No. and n, number; NS, not significant; TLR4: toll-like receptor 4; WBC, white blood cell.

Supplementary Table 2: Univariate analysis of B cell immune profiles in healthy adults, reactive thrombocytosis, polycythemia vera and essential thrombocythemia.

Variables	HA (n=15)	RT (n= 20)	ET (n=30)	PV (n= 15)	ET vs PV	ET vs RT	ET vs HA	PV vs RT	PV vs HA	RT vs HA
					p value	p value	p value	p value	p value	p value
CD69+ B cells (/µL), median (range)	1.0 (0.52-1.97)	1.03 (0.00-8.97)	4.36 (0.33-46.43)	1.51 (0.15-5.94)	<0.001	<0.001	<0.001	NS	NS	NS
CD80+ B cells (/µL), median (range)	3.11 (0.53-5.73)	4.36 (0.89-17.24)	8.99 (1.34-52.12)	3.77 (1.06-18.01)	0.006	0.010	0.001	NS	NS	0.047
CD86+ B cells (/µL), median (range)	5.04 (1.74-9.20)	6.78 (2.80-13.91)	17.65 (1.32-95.99)	5.99 (2.34-25.61)	<0.001	<0.001	<0.001	NS	NS	0.039

Abbreviations: ET, essential thrombocythemia; HA, healthy adults; n, number; NS, not significant; PV, polycythemia vera; RT, reactive thrombocytosis.
*Number=33.
